# Quality of life profile and psychometric properties of the EQ-5D-5L in HIV/AIDS patients

**DOI:** 10.1186/1477-7525-10-132

**Published:** 2012-11-01

**Authors:** Bach Xuan Tran, Arto Ohinmaa, Long Thanh Nguyen

**Affiliations:** 1 School of Public Health, University of Alberta, Alberta, Canada; 2 Institute for Preventive Medicine and Public Health, Hanoi Medical University, Hanoi, Vietnam; 3 Authority of HIV/AIDS Control, Ministry of Health, Hanoi, Vietnam

**Keywords:** EQ-5D, Quality of life, Utility, HIV, Vietnam, Psychometric properties, Antiretroviral treatment

## Abstract

**Objectives:**

We assessed health-related quality of life (HRQOL), its associated factors, and examined measurement properties of the EuroQol - 5 Dimensions - 5 Levels (EQ-5D-5L) in HIV/AIDS patients.

**Methods:**

A cross-sectional multi-site survey was conducted in 1016 patients (age: 35.4 ± 7.0 years; 63.8% male) in three epicenters of Vietnam. Internal consistency reliability, convergent validity, and discriminative validity of the EQ-5D-5L and a visual analogue scale (VAS) were evaluated. Tobit censored regression models were used to identify predictors of HRQOL in HIV/AIDS patients.

**Results:**

The mean EQ-5D-5L single index and VAS were 0.65 (95% Confidence Interval (CI) = 0.63; 0.67) and 70.3 (95% CI = 69.2; 71.5). Cronbach’s alpha of five dimensions was 0.85. EQ-5D-5L has a good convergent validity with VAS (0.73). It discriminated patients at different HIV/AIDS stages, duration of ART, and CD4 cell count. Predictors of poorer HRQOL included being female, lower education level, unemployment, alcohol and drug use, CD4<200 cells/mL, and advanced HIV/AIDS stages.

**Conclusion:**

The EQ-5D-5L has good measurement properties in HIV/AIDS patients and holds potentials for monitoring ART outcomes. Integration of HRQOL measurement using EQ-5D-5L in HIV/AIDS clinical practice could be helpful for economic evaluation of HIV/AIDS interventions.

## Introduction

Health-related quality of life (HRQOL) has become an important indicator to assess outcomes of health interventions, as well as inform patient management and policy development. In HIV research, HRQOL measurement has advanced rapidly in recent years, especially since antiretroviral treatment (ART) services were scaled-up that transformed HIV/AIDS into a chronic illness [1]. Assessments of HRQOL supplements traditional measures of HIV-related morbidity and mortality. HRQOL was found to be associated with physical and clinical characteristics of HIV/AIDS patients, including opportunistic infections, immune status and viral load [2-4]. Moreover, HRQOL includes broader non-medical aspects of patients living with HIV/AIDS, such as psychological, interpersonal, spirituality, and environment [5,6]. Therefore, measurements of HRQOL can better reflect changes in HIV/AIDS patients and inform economic evaluation of HIV/AIDS treatment services.

Basically, HRQOL can be assessed using generic or condition specific measures. Generic measure included health status profile or utility (preference-based) measures that provide a summary of HRQOL. Specific measures are those instruments which focus on problems associated with single disease states, patient groups, or areas of function [7]. There have been a number of instruments, both generic or HIV/AIDS specific, applied in HIV/AIDS patients, for instance, the WHOQOL-HIV, MOS-HIV, EQ-5D, SF-6D [8-11]. While specific measures are sensitive to clinical aspects of HIV/AIDS, they are often in the form of long profiles that might be time-consuming and inconvenient in clinical practice. Generic preference-based measures such as EQ-5D, SF-6D, in contrast, come in a short and convenient form, thus, would be easier to administer in HRQOL measurements [8]. Nonetheless, preference-based measures usually have a high ceiling effect and might be less responsive to monitor changes in HRQOL of patients during ART [12]. Recently, the EuroQOL Group has introduced a 5-level version of the EQ-5D. This promises better measurement properties in terms of improving the instrument’s sensitivity and reducing ceiling effects by increasing the number of severity levels [13,14]. However, since this version is newly introduced, evidence of its improvements in psychometric properties is very limited.

Vietnam has one of the fastest growing HIV epidemics in Asia, which was largely driven by high-risk behaviors, including unsafe sex and drug injection [15,16]. Comprehensive HIV/AIDS care and treatment is a cost-effective intervention recognized in the National HIV/AIDS Strategic Plan [16,17]. Over the last five years, ART services have been rapidly scaled-up, covering 59,000 HIV/AIDS patients, approximately 18 times the ART coverage in 2005. There have been 318 ART clinics established in the country, of those 50% at the district, 48% at the provincial, and 2% at the central level [18]. The expansion and decentralization of ART services promise substantial benefits to HIV/AIDS patients and society. However, at the same time, concerns are raised about the service’s quality [19]. This underscores the importance of monitoring HIV/AIDS treatment outcomes as well as identifying feasible and valid methods of HRQOL assessments. In the present study, we assessed health-related quality of life (HRQOL), its associated factors, and examined measurement properties of the EuroQol - 5 Dimensions - 5 Levels (EQ-5D-5L) in HIV/AIDS patients attending ART clinics in Vietnam.

## Methods

### Study design and participant recruitment

A cross-sectional study was conducted in Hanoi, Hai Phong, and Ho Chi Minh City in 2012. These metropolitans represent different geographical areas with the largest HIV epidemics in Vietnam. Ha Noi is the capital of Vietnam with a population of 6 million, among those, 18,108 people living with HIV. Hai Phong is a port city in northern Vietnam of about 1.8 million citizens, among those, 6930 HIV-positive cases reported. Ho Chi Minh City is the biggest southern metropolitan centre, which has the largest HIV-positive population nationwide with 46,507 cases over 8 million residents [20,21]. Seven hospitals across levels of the health system were purposively selected for the study where 1016 patients were interviewed (17% of the total sample frame). The sample size was allocated 20% for central (the National Hospital for Tropical Diseases), 40% for provincial (Dong Da, Viet Tiep, and Ho Chi Minh City Tropical Diseases Hospitals), and 40% for district clinics (Tu Liem, Le Chan, and Binh Tan district health center). The eligibility criteria for ART in Vietnam during the period of this study included a CD4 count less than 250 cells/mL. Patients with HIV/AIDS, registering for care or treatment at selected sites, and present at the clinics during the period of the survey, were invited to participate in the study. This included both inpatients and outpatients, who came for receiving regular check-ups and ARV medications. Selection of patients was basically convenient until we got a sufficient sample size given the fact that we could not develop a sample frame of HIV/AIDS patients due to confidentiality. The response rate was 85-90% in different clinics.

### Measures and instrument

Face-to-face interviews using a structured questionnaire were carried out by well-trained health workers to collect information about patient’s socioeconomic, HIV-related characteristics, and HRQOL. The socioeconomic characteristics included age, gender, marital status, education level, employment, and income. Household’s monthly income was self-reported by summing up all sources of income from each member in 2011; and then stratified into five quintiles. Health behaviors of interest, including drug use, alcohol use, and adherence to ART, were also self-reported by patients during the interview. We used the Alcohol Use Disorders Identification Test - Consumption (AUDIT-C) to assess alcohol use disorders (AUD) in HIV/AIDS patients [22]. It includes three questions: 1) How often do you have a dink containing alcohol?; 2) How many standard drinks containing alcohol do you have on a typical day?; and 3) How often do you have six or more drinks on one occasion? Responses to each question were assigned a score from 0 to 4 that make the AUDIT-C’s total score falls into a scale of 0–12. Higher scores indicate higher likelihood that the patient’s health and safety are at risk. An AUDIT-C score of (4 or more) in men and (3 or more) in women is considered positive and used for classifying individuals with active AUD. The Vietnamese version of AUDIT instrument was developed and validated [22,23]. Patients were asked if they had ever used opiates. In a longitudinal study of drug users in Vietnam, self-reported drug use was associated with changes in HRQOL [24]. Patients’ adherence to ART over the last 30 days was self-reported on a visual analogue scale (VAS). The threshold for optimal adherence was defined at 95% and above [25]. Clinical characteristic, including HIV/AIDS stages, CD4 cell count, and the duration of ART were extracted from medical records over the past three months.

*The EuroQOL - 5 Dimensions (EQ-5D)* was employed for measuring health-related quality of life of HIV/AIDS patients. It consisted of a weighted sum of five dimensions: Mobility, Self-care, Usual activities, Pain/Discomfort and Anxiety/Depression, which provided a simple descriptive profile and a single index value for health status [26]. The Vietnamese version of the EQ-5D 3-levels has been applied in HIV/AIDS patients as well as general population in Vietnam [27]. We used a recently developed version, the EQ-5D-5L, which includes five-level response options: no problems, slight problems, moderate problems, severe problems, and extreme problems [14]. EQ-5D-5L defined 3125 health states, which were converted into a single index. The Vietnamese version of the EQ-5D was provided by the EuroQOL and culturally adopted in Vietnam [27]. Prior to the survey, three focus group discussions were carried out in researchers, interviewers and HIV/AIDS patients to examine the relevance of five new response options of the EQ-5D-5L. Since population reference of Vietnam was not available, we used the interim scoring for EQ-5D-5L from the cross-walk value set of Thailand [28]. Besides, the EQ-5D-5L also includes a visual analogue scale (EQ-VAS), which recorded the patient’s self-rated health on a 20 cm vertical, visual analogue scale with endpoints (0; 100) labeled ‘the best health you can imagine’ and ‘the worst health you can imagine’. Finally, patients also reported their general HRQOL on a global rating scale of five response options: very poor, poor, moderate, good, and very good.

### Statistical analysis

*Descriptive statistical analysis was* used to describe the socio-demographics and HIV-related characteristics, and EQ-5D-5L health profile of respondents. ANOVA test was used to examine the differences between means of HRQOL of various patient groups.

*Psychometric properties of the EQ-5D-5L measurement*: Internal consistency reliability of measurement was estimated using Cronbach’s alpha. Although EQ-5D consists of multiple dimensions with single items, the use of Cronbach’s alpha for evaluating its reliability has been reported in previous studies [29-32]. Discriminative validity was assessed by testing “a priori” hypotheses that the measure could distinguish patients who were at different clinical groups: (1) HIV/AIDS stages, (2) CD4 cell counts, and (3) Duration of ART; and who had different health behaviors: (4) drug use and (5) alcohol use; and (6) who had suboptimal ART adherence [18,24,27,33-36]. The differences between known groups relative to standard deviation of EQ-5D index and VAS score were estimated for assessing the effect size. Effect size of (0.2-0.5), (0.5-0.8), and (>0.8) indicates small, moderate and large differences in HRQOL. The percentage of respondents at floor and ceiling scores were examined for each dimension of the EQ-5D. The EQ-5D-5L single index was correlated with VAS score and a global rating of health-related quality of life to examine the convergent validity of the measurement.

*Multivariable linear regression* was used to determine factors associated with HRQOL domain’s scores. Since the range of EQ-5D-5L single index and VAS ranged at (−0.452; 1) and (0; 100), these scales were left- and right censored. Censoring from above and below the HRQOL scores did not allow us to measure exactly the values which were higher or lower than the range thresholds. Therefore, in regression analysis, we employed censored regression models (Tobit models) to determined factors associated with EQ-5D-5L single index and VAS [37]. Candidates for multivariate models included those variables that met one of the following three criteria: (1) socio-economic status, (2) biological association with the outcome of interest; and (3) previously shown to be associated with the health-related quality of life among Vietnamese population [18,27,33]. We identified a set of candidate variables, including: (1) patient’s characteristics: gender, age, education, marital status, employment and income; (2) HIV/AIDS-related characteristics: CD4 cell count, HIV stages, and ART; and (3) patient’s adherence to ART, alcohol use, and drug use [18,24,27,33,38]. This set of candidate variables were entered in the full regression models. After that, we applied a stepwise forward selection to determine the reduced model that selected variables based on the log-likelihood ratio test at a p-value < 0.1, and excluded variables at p-values >0.2.

### Ethical consideration

The use of survey data was approved by the Authority of HIV/AIDS Control, Ministry of Health of Vietnam. Ethical approvals granted by the University of Alberta’s Health Research Ethics Board. Written informed consent was obtained from all participants after clearly introduce the survey. Respondents could refuse to participate or withdraw from the interview at any time, and this did not affect their continuation of health care services. Confidentiality was assured using codes of patient’s information, and secured storage was prepared for both paper questionnaires and electronic data set.

## Results

### Socio-demographic and clinical characteristics of respondents

Of 1016 patients interviewed, 63.8% were male, average age was 35.4 (SD=7.0), 45% had high school education and above, and 20.4% had stable jobs. The average monthly income per capita of their households was roughly US$ 100 in 2011. As for HIV-related characteristics, Table 1 shows that more than one-third of respondents were in AIDS stage (37.6%), half of them were symptomatic HIV stage (50%) and only 12.4% were asymptomatic. There were 88.8% patients taking ART, among those, 55.3% have been treated for more than two years. The majority of patients remained low CD4 cell counts, 86.4% had <= 500 cells/mL. A large proportion of the sample were those patients who had a history of drug use (46.1%), and who reported having AUD (30.1%), and who reported suboptimal adherence to ART (24.7%).

**Table 1:** EQ-5D-5L profile of patients by different disease stages

	**Asymptomatic**		**Symptomatic**		**AIDS**		**All**	
	**N**	**%**	**N**	**%**	**N**	**%**	**N**	**%**
**MOBILITY**								
- Unable to walk about	2	1.6	10	2.0	21	5.5	33	3.3
- Severe problems	7	5.6	24	4.7	22	5.8	53	5.2
- Moderate problems	9	7.1	49	9.7	34	8.9	92	9.1
- Slight problems	26	20.6	149	29.3	105	27.5	280	27.6
- No problems	82	65.1	276	54.3	200	52.4	558	54.9
**SELF-CARE**								
- Unable to wash or dress myself	1	0.8	3	0.6	17	4.5	21	2.1
- Severe problems			15	3.0	11	2.9	26	2.6
- Moderate problems	1	0.8	22	4.3	18	4.7	41	4.0
- Slight problems	11	8.7	60	11.8	46	12.0	117	11.5
- No problems	113	89.7	408	80.3	290	75.9	811	79.8
**USUAL ACTIVITIES**								
- Unable to do	2	1.6	8	1.6	24	6.3	34	3.4
- Severe problems	3	2.4	17	3.4	20	5.2	40	3.9
- Moderate problems	5	4.0	37	7.3	23	6.0	65	6.4
- Slight problems	21	16.7	119	23.4	81	21.2	221	21.8
- No problems	95	75.4	327	64.4	234	61.3	656	64.6
**PAIN/DISCOMFORT**								
- Extreme pain	2	1.6	5	1.0	17	4.5	24	2.4
- Severe pain			19	3.7	18	4.7	37	3.6
- Moderate pain	5	4.0	52	10.2	38	10.0	95	9.4
- Slight pain	48	38.1	241	47.4	146	38.2	435	42.8
- No pain	71	56.4	191	37.6	163	42.7	425	41.8
**ANXIETY/DEPRESSION**								
- Extremely	5	4.0	19	3.7	22	5.8	46	4.5
- Severely	6	4.8	49	9.7	32	8.4	87	8.6
- Moderately	17	13.5	96	18.9	59	15.5	172	16.9
- Slightly	50	39.7	225	44.3	157	41.1	432	42.5
- Not anxious or depressed	48	38.1	119	23.4	112	29.3	279	27.5

### Quality profile and psychometric properties of the EQ-5D-5L in HIV/AIDS patients

Table 1 describes the HRQOL profile of HIV/AIDS patients by different disease stages. We found a lower proportion of having no problems across all 5 HRQOL dimensions in patients at later disease stages compared to asymptomatic patients. Overall, it was the highest in “Self-care” (79.8%) and the lowest in “Anxiety/Depression” (27.5%). The EQ-5D-5L showed a small proportion of respondents reported “very poor” status; the highest was 6.3% in “Usual activities”. There were 18.8% patients reported having no problem in all five dimensions. The mean EQ-5D index and VAS were 0.65 (SD= 0.27) and 70.3 (SD=19), respectively.

Table 2 shows the “known groups” validity of the EQ-5D-5L, both single index and VAS score, in discriminating patients at different HIV/AIDS stages, CD4 cell count groups, duration of ART, and having AUD. Differences between the highest and lowest HRQOL of these known groups ranged from 0.2 (AUDIT-positive) to 0.6 (CD4 counts), indicating “small” to “moderate” effect sizes. In addition, the VAS could distinguish IDU and non-IDU patients at a level of significance p=0.09; however, the effect size was trivial. The convergent validity of EQ-5D-5L index score with VAS was good (0.73), and with the global rating of HRQOL was fair (0.36). Cronbach’s alpha of five dimensions was 0.85.

**Table 2:** HRQOL of different patient groups and discriminative validity of the EQ-5D-5L

	**N**	**%**	**EQ-5D Index**				**VAS**			
			**Mean**			**p-value**	**Mean**			**p-value**
**All patients**	1016	100.0	0.65	0.63	0.67		70.34	69.17	71.51	
**HIV Stages**										
- Asymptomatic	126	12.4	0.73	0.69	0.77	<0.01	76.67	74.14	79.20	<0.01
- Symptomatic	508	50.0	0.65	0.63	0.67		70.72	69.15	72.29	
- AIDS	382	37.6	0.62	0.59	0.66		67.79	65.66	69.91	
**CD4**										
<=200	249	31.1	0.58	0.54	0.61	<0.01	64.47	61.80	67.14	<0.01
200<cd4<=350	249	31.1	0.66	0.62	0.69		71.62	69.43	73.81	
350<cd4<=500	194	24.2	0.73	0.70	0.75		75.57	73.48	77.65	
>500	109	13.6	0.69	0.65	0.74		76.22	73.20	79.24	
**Duration of ART**										
Not yet	114	11.2	0.64	0.59	0.69	<0.01	68.15	64.50	71.80	<0.01
<=1 yr	196	19.3	0.58	0.54	0.62		65.53	62.57	68.50	
1; <=2	144	14.2	0.65	0.61	0.69		71.54	68.58	74.49	
2; <=4	270	26.6	0.68	0.65	0.71		71.76	69.56	73.95	
4; <=7	292	28.7	0.68	0.64	0.71		72.52	70.44	74.60	
**IDU**										
- No	548	53.9	0.66	0.63	0.68	0.48	71.29	69.72	72.85	0.09
- Yes	468	46.1	0.64	0.62	0.67		69.24	67.46	71.01	
**AUDIT-positive**										
- No	710	69.9	0.67	0.65	0.69	<0.01	71.73	70.40	73.07	<0.01
- Yes	306	30.1	0.60	0.57	0.63		67.12	64.78	69.47	
**Optimal ART adherence**										
- No	660	75.3	0.65	0.63	0.67	0.91	71.15	69.69	72.62	0.47
- Yes	217	24.7	0.65	0.62	0.69		70.09	67.73	72.44	

### Factors associated with HRQOL in HIV/AIDS patients

The mean of EQ-5D-5L single index and VAS was 0.65 (95% Confidence Interval (CI) = 0.63; 0.67) and 70.3 (95% CI = 69.2; 71.5), respectively (Table 2). Table 3 presents associations between patient’s characteristics and EQ-5D-5L single index and VAS scores in reduced multivariate models. Patients at the provincial level reported lower HRQOL than whom attending central clinics. Socio-demographic characteristics of respondents negatively correlated with EQ-5D-5L single index and VAS score included female sex, lower education attainment, and unemployment. Later stages of HIV/AIDS infections and deterioration in immune function were associated with poorer HRQOL. Over the course of ART, VAS score was lower in the first year of ART compared to those not-yet taking it; meanwhile, EQ-5D-5L single index was stable and significantly increased after the 2^nd^ year of treatment. Besides, health behaviors also predicted HRQOL in HIV/AIDS patients. Drug users reported lower VAS score than non-drug users. Patients who had AUD reported lower score in both VAS and EQ-5D-5L single index than others.

**Table 3:** Determinants of HRQOL in HIV/AIDS patients using EQ-5D-5L and VAS

	**EQ-5D index (−0.452; 1)**		**VAS (0; 100)**	
	**Coef.**	**95% CI**	**Coef.**	**95% CI**
**Level of Health System** (^a^ Central)				
- Provincial	−0.11***	(−0.16; -0.66)	−7.8***	(−10.5; -5.0)
**Gender** (^a^ Male)				
- Female			−4.5***	(−7.5; -1.6)
**Education** (^a^ Below high school)				
- High school and above	0.06**	(0.01; 0.10)	3.8***	(1.0; 6.5)
**Marital status** (^a^ Single)				
- Live with spouse/partner			−2.0	(−4.9; 0.9)
**Employment** (^a^ Unemployment)				
- Free-lancer ^b^	0.12***	(0.06; 0.19)	9.6***	(5.9; 13.4)
- Stable jobs	0.09**	(0.02; 0.17)	9.1***	(4.7; 13.6)
- Others	0.13***	(0.04; 0.23)	11.3***	(5.8; 16.8)
**Income quintiles** (^a^ Poorest)				
- Richest	0.05	(−0.01; 0.11)		
**HIV/AIDS Stages** (^a^ Asymptomatic)				
- Symptomatic	−0.2***	(−0.3; -0.1)	−7.1***	(−12.0; -2.2)
- AIDS	−0.1***	(−0.2; -0.1)	−8.1***	(−13.2; -3.0)
**CD4 cell count** (^a^ Less than 200)				
200<cd4<=350	0.06**	(0.00; 0.12)	4.5**	(1.0; 8.0)
350<cd4<=500	0.15***	(0.08; 0.22)	8.8***	(4.9; 12.7)
>500	0.07*	(−0.01; 0.15)	8.4***	(3.7; 13.0)
**Duration of ART** (^a^ Not yet)				
<=1 yr			−4.8***	(−8.5; -1.2)
1-- <=2 yrs				
2-- <=4 yrs	0.07**	(0.01; 0.14)		
4-- <=7 yrs	0.08**	(0.01; 0.14)		
**History of drug use**			−3.9***	(−6.8; -1.1)
**Having AUD**	−0.06**	(−0.11; -0.01)	−3.1**	(−6.2; -0.1)
Constant	0.65***	(0.55; 0.76)	74.0***	(67.3; 80.8)

*** p<0.01, ** p<0.05, * p<0.1, ^a^ Reference group.

^b^ Self-employed, non-salary basis.

## Discussion

We assessed the HRQOL of HIV/AIDS patients attending ART clinics using the EQ-5D-5L profile, single index, and VAS, and determined the association of socioeconomic and clinical characteristics and HRQOL. The instrument showed an excellent reliability and good discriminative validity. We found a high proportion in Anxiety/Depression and Pain/Discomfort among HIV/AIDS patients who were taking ART. The health utility of patients was 0.65 and 0.70 for EQ-5D-5L single index and VAS, respectively. Gender, education, and employment were significantly associated with HRQOL. Advanced HIV/AIDS patients, poor immune status, having AUD and drug use, were associated with poorer HRQOL.

This is the first study examining psychometric properties of the new version - EQ-5D-5L in HIV/AIDS patients. It showed that the reliability was 0.85, which was close to the threshold of 0.90, indicating the potential use of EQ-5D-5L for measuring HRQOL at an individual level. In the literature, applications of the EQ-5D-3L in HIV/AIDS showed its convergent validity with the MOS-HIV, and discriminative validity with AIDS-defining events, disease severity (CD4, viral load, and HIV/AIDS stages) [35,36,39]. We observed the same validity of the EQ-5D-5L in discriminating patients at different CD4 groups, and HIV/AIDS stages. In addition, we found that EQ-5D-5L could distinguish patients at different clinically meaningful duration of ART. Another important characteristic of the EQ-5D-5L was that it had a smaller ceiling effect and average HRQOL score of the HIV/AIDS population than the previous 3-level version [27]. Therefore, there will be room for assessing the improvements of HRQOL overtime if one would like to use this tool for longitudinal assessments. Consequently, findings of this study contribute to the cumulative evidence of measurement properties of the EQ-5D instruments in HIV/AIDS population that may inform the selection of measures in both cross-sectional and follow-up designs.

Compared with previous studies, we found some similar influential factors of HRQOL in HIV/AIDS patients, for example, employment was positively associated with HRQOL [27,33]. As for the duration of ART, there was a consistent finding that patients might experience HRQOL reduction during the ART compared to those not-yet-eligible for ART [27]. This could be due to the immune deterioration and negative impact of ART side effects. Moreover, we found that changes in HRQOL during ART were non-linear: it decreased within the first year of ART, but then increased afterwards. We also confirmed the negative impacts of alcohol use and drug use on HRQOL outcomes of ART for HIV/AIDS patients [24,27]. In practice, both alcohol and drug use are known to be associated with delayed access to health care, suboptimal adherence, more severe co-morbidities, and poorer outcomes of ART [22,24].

This study has helpful implications for both HRQOL assessments and ART services. The measurement properties of the EQ-5D-5L holds potentials in monitoring changes in HRQOL, which are associated with meaningful clinical indicators, such as, CD4 thresholds, HIV/AIDS progression, or responses to ART treatment. Moreover, it helps identify areas for interventions to improve the health outcomes of patients. In this study, we observed a very high prevalence of reported problems in Anxiety/Depression that suggests the necessity of psychological support during ART for HIV/AIDS patients. As for the patient management, the 1^st^ year ART is an important period when we observed a significant reduction in HRQOL. During this period, patients had to adapt with the strict compliance, residual opportunistic infections, and side effects of antiretroviral medications. The adherence should also be maintained during more stable periods of ART afterwards. In the Vietnamese settings, stigma and discrimination were found to be a significant barrier that affected adherence to and HRQOL outcomes of ART [27]. The reduction in HRQOL observed in women suggests that gender-specific impact mitigation and support interventions should be in place. Previous works identified that peer’s support, vocational training, job referrals, and microfinance are potential interventions to support women with HIV/AIDS [27,33,40-42].

The strengths of this study include the involvement of patients in three epicenters across levels of health systems and geographical areas of Vietnam. In addition, we employed the Thailand tariff for deriving the EQ-5D-5L index, which has close culture and other characteristics with Vietnam. Besides, there were some limitations that should be acknowledged. First, we did not have viral load assessments, a gold standard in monitoring ART outcomes, since viral load tests are costly and not regularly assessed for all HIV/AIDS patients in Vietnam. Second, tests of convergent validity compared only the measures derived from the same EQ-5D instrument. Moreover, the cross-sectional design might be less capable for causal inference as well as for assessing the responsiveness of the EQ-5D-5L in this measurement. In addition, patients at clinics were selected conveniently making the sample not representative for the population of HIV/AIDS patients and limiting the generalizability of study findings.

In conclusion, the EQ-5D-5L showed good properties in measuring HRQOL of HIV/AIDS patients and has potentials for monitoring ART outcomes. Integration of HRQOL measurement using EQ-5D-5L in HIV/AIDS clinical practice could be helpful for economic evaluation of HIV/AIDS interventions. Further research to develop a scoring system of the Vietnamese population is recommended.

## Competing interests

The authors declare no funding support or conflicts of interest.

## Authors’ contributions

BXT and LTN designed and implemented the survey. BXT and AO analyzed the data. BXT, AO, and LTN wrote the manuscript. All authors read and approved the final manuscript.
